# Disulfide bond mapping of Pfs25, a recombinant malaria transmission blocking vaccine candidate

**DOI:** 10.1016/j.ab.2017.11.009

**Published:** 2018-02-01

**Authors:** Shwu-Maan Lee, Jordan Plieskatt, C. Richter King

**Affiliations:** PATH Malaria Vaccine Initiative (MVI), 455 Massachusetts Avenue NW, Suite 1000, Washington, DC 20001-2621, USA

**Keywords:** Pfs25, *Plasmodium falciparum*, Malaria, Baculovirus, Disulfide

## Abstract

A liquid chromatography tandem-mass spectrometry method was developed to map the eleven disulfide bonds in Pfs25, a malaria transmission-blocking vaccine candidate. The compact and complex nature of Pfs25 has led to difficulties in prior peptide mapping efforts. Here, we report confirmation of proper disulfide pairing of a recombinant Pfs25, by optimizing denaturation and digestion with trypsin/Lys-C. The digested peptides were separated by reversed phase HPLC to obtain the peptide map and elucidate the disulfide linkages. MS^E^ fragmentation confirmed the digested peptides and disulfide bonds. The eleven disulfide bonds and locations matched the predicted Pvs25 crystal structure, a Pfs25 homologue.

## Graphical abstract

**Figure:**
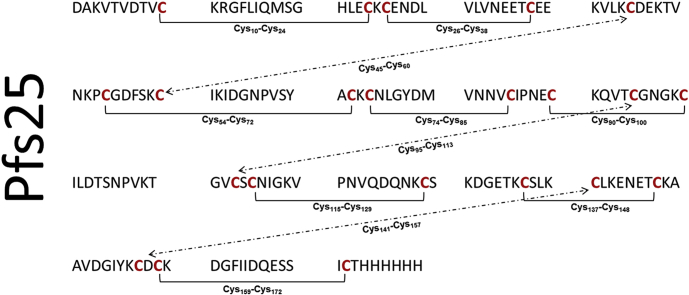

The *Plasmodium falciparum* surface protein Pfs25 [1], [2], [3], has long been presented as a potential transmission-blocking vaccine (TBV) candidate for parasite elimination [4], [5], [6] and control of malaria. The expression, purification, and characterization of the Pfs25 antigen has also been well presented from various heterologous expression systems including yeast [7], [8], [9], *E. coli* [10], plant [11], algae [12], and recently baculovirus [13]. An important consideration in the selection of expression system and production of recombinant proteins is maintaining the native presentation and conformation of the molecule to raise antibodies that would recognize (and interfere) with those proteins found on the parasite itself. The proper disulfide bond formation of the molecule would be critical not only for proper protein folding but also for the stabilization of the molecule including its tertiary structure. This is especially true for the development of TBV candidates, where the native proteins are often complex, disulfide-rich molecules, where native conformation would be required to elicit the proper antibody response to block the parasite [14]. The importance of disulfide pairing has been demonstrated in a reduction alkylation experiment, which abolished the functional activity of Pfs25 [13],

However, methods to effectively map the eleven disulfide bonds present in Pfs25 and confirm proper pairing had yet to be reported, an important step in the development of recombinant vaccine antigens. The effort by Gregory et al. [12] was the last reported attempt to disulfide bond map Pfs25. When the disulfide bonds from algae Pfs25 were identified and compared to the yeast-produced homologue Pvs25 [15], they found that disulfide bonds one, four, and six were intact; however, disulfide bonds two, seven, eight, ten, and eleven may not have been completely formed [12]. The complex, compact and unusual structure of Pfs25 [2], [16] may have contributed to difficulties in prior attempts to analyze the disulfide pairs of Pfs25. Here, we report an optimized digestion and analysis methodology to confirm the 22 cysteines are arranged in 11 disulfide pairs in our baculovirus-expressed Pfs25 [13].

In this study, we first utilized a plant-produced Pfs25 [11] to optimize the digestion and analysis methodology. The plant based Pfs25 was selected for method development, as this protein had served as reference standard during the development of baculovirus derived Pfs25 [13], including its efficacy and transmission-reducing activity previously demonstrated [11], [13]. Given that the molecule was previously established (both in biochemical and *in vivo*) characterization prior to the development of our baculovirus Pfs25, it was thought to serve as a suitable control for method development while the baculovirus Pfs25 was undergoing process development. Initially TCEP (Tris-2 carboxyethyl phosphine HCl) followed by Trypsin and Glu-C digestion was employed. Given difficulties, due to the compact nature of the molecule [2], [16], a portion of the structure may not have been accessible to proteolytic digestion and attempts were made to optimize digestion using Lys-C (data not shown). The enzyme mixture (Trypsin/Lys-C), combined with overnight digest, followed by an additional enzymatic digestion the subsequent day, significantly improved digestion and resulted in a higher intensity for the MS/MS (MS^E^) fragments (data not shown).

The optimized denaturation and digestion was applied to the baculovirus-expressed Pfs25, which we previously reported as a pure, stable, and well characterized protein with no free thiol as determined by Measure iT [13]. A total of 100 μL Pfs25 (1 mg/mL in 20 mM HEPES, 150 mM NaCl, pH 7.0 [13]) was denatured at 80 °C for 30 min then diluted with 100 μL of 1 M Tris-HCl pH 8.0 buffer (Sigma-Aldrich; St. Louis, MO). A total of 20 μL trypsin/Lys-C mix (Promega; Madison, WI) at 1 μg/μL was added and the sample incubated at 37 °C overnight. An additional 20 μL of trypsin/Lys-C was added the following day and the sample further incubated at 37 °C for 3–4 h.

The chromatographic separation of digest peptides was performed using a 2695 Separations Module (Waters Corporation; Milford MA) with a 2489 UV/Vis Detector (Waters Corporation; Milford, MA) set at 214 nm. An XBridge (Waters Corporation; Milford, MA) BEH 300 C18 (2.1 × 250 mm, 5 μm) was used at a column temperature of 37 °C and peptides eluted using a gradient program that included 0.1% Triflouroacetic acid (TFA) in purified water (Mobile Phase A) and 0.1% TFA in acetonitrile (Mobile Phase B). The gradient consisted of: 2% B for 3 min increased to 40% B from 3 to 90 min, increased to 90% B from 90 to 110 min, held at to 90% B from 110 to 112 min, then returned to initial conditions from 112 to 113 min, and held 7 min (Table 1).

**Table 1:** Method for separation and ESI-MS conditions for Pfs25.

HPLC Method	Parameter
HPLC/MS System	Waters Alliance 2695 Waters Q-ToF Premier Mass
Mobile Phase A	0.1% TFA^a^ in water
Mobile Phase B	0.1% TFA^a^ in ACN^b^
Flow Rate	0.2 mL/min
Column	Waters XBridge BEH 300 C18, 2.1 × 250 mm, 5 μm
Column Temperature	37 °C
Auto sampler Temperature	5 °C
Injector Volume	100 μL
Detector Wavelength	214 nm
Run Time	120 min

	Time (Min)	% Mobile Phase A	% Mobile Phase B
Gradient Program	0	98	2
	3	98	2
	90	60	40
	110	10	90
	112	10	90
	113	98	2
	120	98	2

ESI-MS Method	Parameter
Ionization	Electrospray Positive V Mode
M/Z Scan Range	100-3000 Da
Scan Time	3-112 min
Switch Valve	0-3 min and 112–120 min to waste
	3-112 min to mass spectrometer
Capillary Voltage	3.0 kV
Sample Cone Voltage	30.0 V
Source Temperature	120 °C
Desolvation Temp.	250 °C
MS Condition	MS^E^
Low Collision Energy	15 eV
Elevated Collision Energy	Ramping from 20 to 90 eV
Scan Time	1.5 s

a Trifluoroacetic acid.

b Acetonitrile.

For mass spectrometric (MS) analysis, a QTOF Premier mass spectrometer (Waters Corporation; Milford, MA) equipped with an electrospray source and lock spray run in positive mode (ES+) was used. MS data was acquired in MS^E^ mode using MassLynx v4.1 (Waters Corporation; Milford, MA). Tuning mix solution (Agilent ESI-L Tuning Mix, Cat# G1969-85000; diluted 1:10 in acetonitrile) was infused into the ion source at a flow rate of 1–15 μL/min in the reference channel at 10 s intervals. Full conditions are presented in Table 1.

The mass spectral data was analyzed using BiopharmaLynx 1.3 (Waters Corporation; Milford, MA). The theoretical disulfide bonds were entered into the sequence editor and observed mass spectral peaks compared to the theoretical digests. MassLynx was utilized to validate the observed peptide linkage mass (precursor ion) and BiopharmaLynx was used to confirm disulfide bond linkages by evaluating the observed MS^E^ fragmentation patterns (product ion) against the theoretical patterns based on the sequence and disulfide bonds linked in the editor. The BiopharmaLynx data analysis included an evaluation of scrambled disulfide bonds.

Localization of the disulfide bonds in Pfs25 was accomplished by comparing the accurate mass results for the trypsin/Lys-C peptide map of the protein to theoretical masses for the disulfide linked peptides. Additionally, the peptides were fragmented using the MS^E^ function of MassLynx. BiopharmaLynx was further used to scan for disulfide bond linkages that were not in the expected locations. Pfs25 contained eleven disulfide bonds which were proposed to link the following cysteine residues: Cys_10_-Cys_24_, Cys_26_-Cys_38_, Cys_45_-Cys_60_, Cys_54_-Cys_72_, Cys_74_-Cys_85_, Cys_90_-Cys_100_, Cys_95_-Cys_113_, Cys_115_-Cys_129_, Cys_137_-Cys_148_, Cys_141_-Cys_157_, and Cys_159_-Cys_172_. The theoretical masses of the digested peptides are also presented (See Table 1 in Ref. [17]) along with disulfide bond locations (see Table 2 in Ref. [17]). Fig. 1 serve to provide labels for these disulfide bonds that will be used in further discussion of their elucidation. When tryptic peptides are linked by a disulfide bond, they were represented in a format such as T2+T4 to indicate tryptic peptides two and four were linked by a disulfide bond. Mass error was defined as the absolute value of the difference between the theoretical and observed masses divided by the theoretical mass and then multiplied by 10^6^ (ppm). In brief, all eleven disulfide bonds were detected and identified by the MS/MS data.

**Fig. 1:**
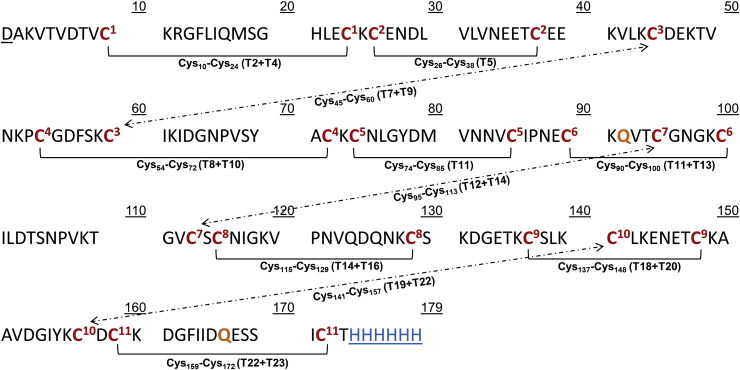
**Pfs25 disulfide pairing as experimentally confirmed by LC-MS/MS**. Amino acid sequence of baculovirus-expressed Pfs25 indicating disulfide bond locations and cysteine pairing. Cysteine locations are indicated in **red**, with disulfide pairs indicated by superscript number and brackets (or arrows) as experimentally confirmed in this study. Digested peptides are indicated by **T#**. Mutations from asparagine to glutamine are indicated in **orange**. A residual aspartic acid (underline) on the N-terminus is from the Honeybee Melittin secretion signal sequence. C-terminal six histidine affinity tag indicated by (Blue underline).

Disulfide bond (**SS1 Cys**_**10**_**-Cys**_**24**_) linked peptides T2 and T4 with a theoretical peptide mass of 2323.1375 Da and an observed mass of 2323.1152 (m/z 775.3790 for the 3^+^ charge state). Twenty fragment ions supported the elucidation of disulfide bond SS1 (See Table 3, Fig. 1 in Ref. [17]).

Disulfide bond (**SS2 Cys**_**26**_**-Cys**_**38**_) was an internal linkage of peptide T5 with a theoretical mass of 1863.7866 Da and an observed mass of 1863.7752 Da (m/z 932.8949 for the 2^+^ charge state). Thirty-six fragment ions supported the elucidation of disulfide bond SS2 (See Table 4, Fig. 2 in Ref. [17]).

Disulfide bond (**SS3 *Cys***_***45***_***-Cys***_***60***_) linked peptides T7 and T9 with a theoretical mass of 853.3674 Da and an observed mass of 853.3656 Da (m/z 854.3729 for the 1^+^ charge state). Six fragment ions supported the elucidation of disulfide bond SS3 (See Table 5, Figure 3 in Ref. [17]).

Disulfide bond (**SS4 *Cys***_***54***_***-Cys***_***72***_) linked peptides T8 and T10 with a theoretical mass of 2358.0670 Da and an observed mass of 2358.0799 Da (m/z 787.0339 for the 3^+^ charge state). Forty-five fragment ions supported the elucidation of disulfide bond SS4 (See Table 6, Figure 4 in Ref. [17]).

Disulfide bond (**SS5 *Cys***_***74***_***-Cys***_***85***_) was an internal disulfide bond of peptide T11 and (**SS6 *Cys***_***90***_***-Cys***_***100***_) linked peptides T11 and T13. The theoretical mass of this peptide was 3112.3796 Da and an observed mass of 3112.3534 Da reported herein (m/z 1038.4584 for the 3^+^charge state). Four fragment ions were consistent with the internal SS5 and 33 fragment ions supported SS6. An additional 44 fragment ions supported the combined linkage of SS5 and SS6 (See Table 7, Figure 5 in Ref. [17]).

Disulfide bond (**SS7 *Cys***_***95***_***-Cys***_***113***_) linked peptides T12 and T14 and (**SS8 *Cys***_***115***_***-Cys***_***129***_) linked peptides T14 and T16, resulting in three peptide linkages in the digest. The theoretical mass for the peptide linking T12, T14, and T16 was 2117.9326 Da and an observed mass of 2117.9182 Da reported herein (m/z 706.9800 for the 3^+^ charge state). Three fragment ions were specific to the linkage between T12 and T14 and confirmed SS7. Four fragment ions were specific to the linkage between T14 and T16 and confirmed SS8. An additional 21 fragment ions were consistent with the linkage of T12, T14 and T16 (See Table 8, Figure 6 in Ref. [17]).

Disulfide bond (**SS9 *Cys***_***137***_***-Cys***_***148***_) linked peptides T18 and T20 with a theoretical mass of 1169.5056 Da and an observed mass of 1169.4970 Da (m/z 585.7558 for the 2^+^ charge state). Twenty-five fragment ions supported the elucidation of disulfide bond SS9 (See Table 9, Figure 7 in Ref. [17]).

Disulfide bond (**SS10 *Cys***_***141***_***-Cys***_***157***_) linked peptides T19 to T22 and disulfide bond (**SS11 *Cys***_***159***_***-Cys***_***172***_) linked peptide T22 to T23. The theoretical mass for this tri-peptide was 3074.3003 Da and an observed mass of 3074.2795 Da reported herein (m/z 1025.7671 for the 3^+^ charge state). Eleven fragment ions were specific to the linkage of T19 and T22 and confirmed SS10. Three fragment ions were specific to the linkage between peptides T22 and T23 and confirmed SS11. An additional 32 fragment ions were consistent with the linkage of T19, T22 and T23 (See Table 10, Figure 8 in Ref. [17]).

In this study, we described a novel methodology that supported elucidation of the complex disulfide bonding pattern of Pfs25. These data are consistent with our previous report that the final purified protein adopted a fully oxidized form, with all 22 cysteines arranged in disulfide pairs [13]. Further, the data is consistent with the predicted disulfide bond formation for Pfs25 based on x-ray crystallography analysis of the homologue, Pvs25 [15].

While the transmission-reducing efficacy of Pfs25 has been demonstrated in a variety of studies, including algae [12] initially with Freund's adjuvant (and later with four additional adjuvants including aluminum hydroxide) [18] and our own [13] baculovirus Pfs25 with ISA720, the two were not directly compared in a single study with harmonized doses, routes and animal models. Therefore, we cannot extrapolate the importance of proper disulfide bond formation in eliciting transmission-reducing activity, but have previously shown that when disulfide bonds are broken, transmission-reducing activity is abolished [13].

This analysis is the first known and reported disulfide mapping of a recombinant Pfs25 with proper bond formation (according to the Pvs25 structure) and provides a methodology that may be applicable to other complex, heavily disulfide-linked *Plasmodium* proteins.

## Funding

The work was supported by the Bill & Melinda Gates Foundation. The views expressed herein are solely those of the authors and do not necessarily reflect the views of the Foundation.

## Conflict of interest disclosure

None. The authors' declare they have no competing interests.
